# T Cell Fitness and Autologous CAR T Cell Therapy in Haematologic Malignancy

**DOI:** 10.3389/fimmu.2021.780442

**Published:** 2021-11-25

**Authors:** Palak H. Mehta, Salvatore Fiorenza, Rachel M. Koldej, Anthony Jaworowski, David S. Ritchie, Kylie M. Quinn

**Affiliations:** ^1^ School of Health and Biomedical Sciences, Royal Melbourne Institute of Technology (RMIT) University, Bundoora, VIC, Australia; ^2^ Clinical Research Division, Fred Hutchinson Cancer Research Center, Seattle, WA, United States; ^3^ Australian Cancer Research Foundation (ACRF) Translational Laboratory, Royal Melbourne Hospital, Melbourne, VIC, Australia; ^4^ Department of Medicine, University of Melbourne, Melbourne, VIC, Australia; ^5^ Department of Biochemistry, Biomedicine Discovery Institute, Monash University, Clayton, VIC, Australia

**Keywords:** T cell fitness, CAR T cell, haematological cancer, ageing, inflammation, kinase inhibitor

## Abstract

A range of emerging therapeutic approaches for the treatment of cancer aim to induce or augment endogenous T cell responses. Chimeric antigen receptor (CAR) T cell therapy (CTT) is one such approach that utilises the patient’s own T cells, engineered *ex vivo* to target cell surface antigens, to eliminate haematological malignancies. Despite mediating high rates of responses in some clinical trials, this approach can be limited by dysfunctional T cells if they are present at high frequencies either in the starting material from the patient or the CAR T cell product. The fitness of an individual’s T cells, driven by age, chronic infection, disease burden and cancer treatment, is therefore likely to be a crucial limiting factor of CTT. Currently, T cell dysfunction and its impact on CTT is not specifically quantified when patients are considering the therapy. Here, we review our current understanding of T cell fitness for CTT, how fitness may be impacted by age, chronic infection, malignancy, and treatment. Finally, we explore options to specifically tailor clinical decision-making and the CTT protocol for patients with more extensive dysfunction to improve treatment efficacy. A greater understanding of T cell fitness throughout a patient’s treatment course could ultimately be used to identify patients likely to achieve favourable CTT outcomes and improve methods for T cell collection and CTT delivery.

## Introduction

Chimeric antigen receptor (CAR) T cell therapy (CTT) is a potent and relatively new therapy for haematological cancers (1–8). A CAR is essentially a synthetic receptor containing an extracellular single-chain variable fragment antibody domain specific for a tumour associated antigen (TAA) linked directly to intracellular T cell signalling domains. Current commercially manufactured CARs utilise CD3ζ immuno-tyrosine activation motifs combined with co-stimulatory molecule signalling domains from either CD28 or 41BB. CAR T cells can be generated by transducing a patient’s T cells with the CAR transgene, enabling activation and effector functions upon encountering cognate TAA. This enables CAR T cells to eliminate TAA-expressing cells independently of major histocompatibility complex-mediated TAA presentation (9).

**Figure 1:**
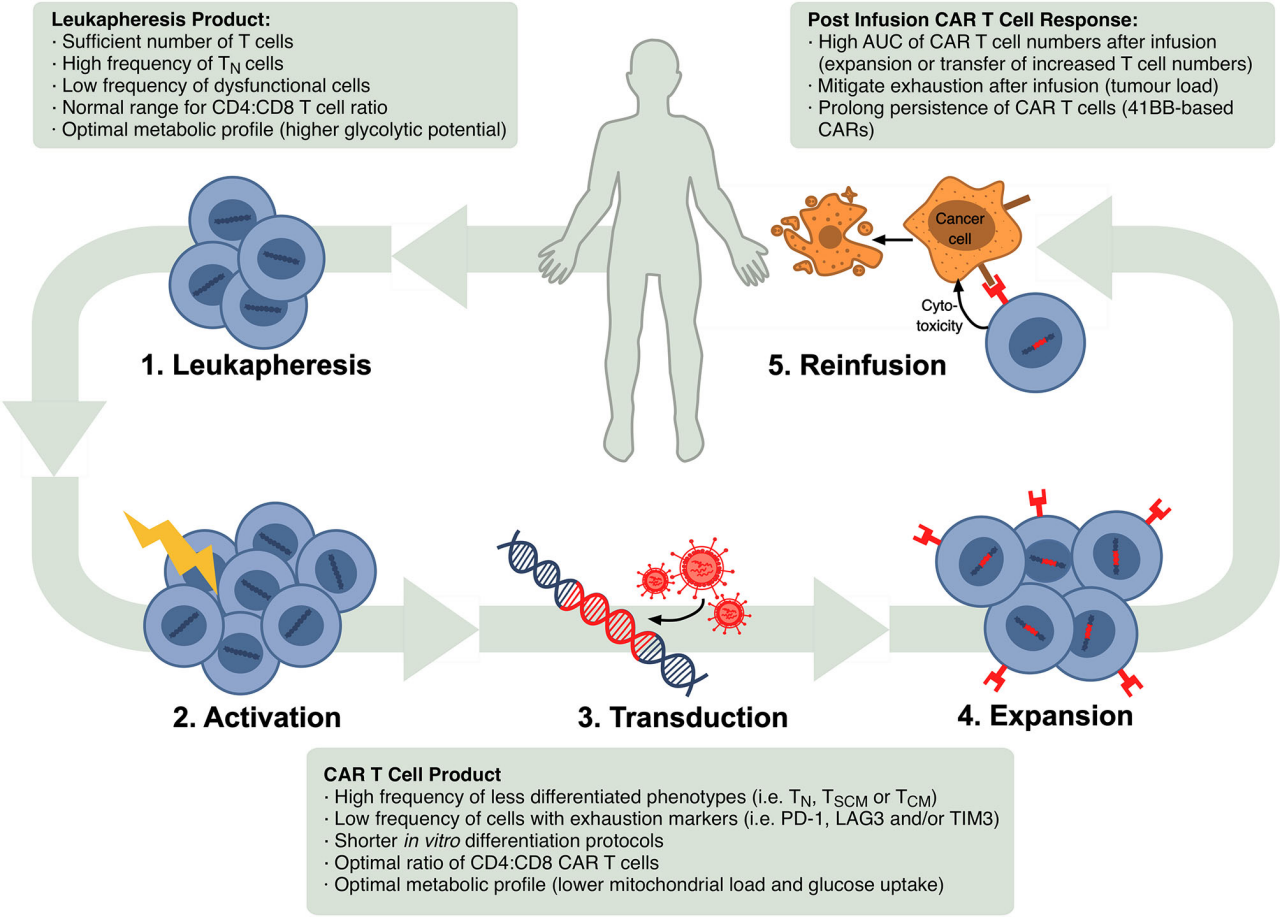
Schematic of steps in the autologous CTT protocol and markers that have been confirmed or are possible correlates of CTT efficacy in the leukapheresis product, CAR T cell product and after re-infusion. In CTT, 1) leukocytes are isolated from the patient’s blood *via* leukapheresis, 2) T cells are activated *in vitro*, 3) activated T cells are stably transduced to express the CAR, commonly using a lentiviral or retroviral vector, 4) CAR-expressing T cells are expanded and 5) infused back into the patient where they can target and eliminate malignant cells.

Autologous CTT progresses in several steps as shown in **Figure 1**. Patients are generally given lymphocyte-depleting chemotherapy prior to the infusion of CAR T cell product. This promotes expansion and activity of CAR T cells by increasing circulating cytokines, such as interleukin (IL)-15 and IL-7 (10, 11), and depleting endogenous myeloid derived suppressor cells and T regulatory cells that might limit CAR T cell responses (12, 13).

Early trials of CTT highlighted its clinical potential, with complete response (CR) rates of 70%-90% observed in trials with patients with adult and paediatric B cell acute lymphocytic leukaemia (ALL) (5, 14–17). However, lower response rates have been observed in trials and real-world studies for other malignancies, including 40-60% in relapsed/refractory (R/R) Non-Hodgkin lymphoma (NHL) (18), ~33% in multiple myeloma (MM) (8) and ~57% in chronic lymphocytic leukaemia (CLL) (1, 19). The disease biology is very different between these patient groups, but NHL, MM and CLL patients also tend to be heavily pre-treated and older. Increased age and certain treatments have been reported to compromise T cell immunity (20, 21) and these factors are also predicted to reduce the quality and fitness of T cells used for CTT.

T cell fitness for CTT can be defined as the ability of a T cell to generate a CAR-mediated immune response that mediates elimination of malignant cells and durable protection from relapsed malignancy. As reviewed below, certain T cell phenotypes and functions in patient peripheral blood mononuclear cells (PBMCs), leukapheresis product and CAR T cell product have been associated with superior patient outcomes with CTT. However, many of these same phenotypes and functions are known to be significantly impacted by age, tumour burden and conventional cancer treatments. Accordingly, we highlight that development of a metric of T cell fitness prior to therapy that captures changes with patient age, disease burden and treatment status will be essential for physicians to advise potential patients and navigate a hierarchy of more tailored treatment options. An understanding of the biology driving these age-, disease- and treatment-related changes in T cell fitness will also enable the design of future CTT regimens optimised for older, heavily treated patients.

CTT is a breakthrough for treatment of haematological cancer, but it can still be improved upon. This review is an opportunity to refine our understanding of biomarkers of CTT efficacy and define T cell fitness as a key driving force behind this efficacy. Ultimately, our perspective is that an assessment of intrinsic T cell fitness will enable more meaningful personalisation of CTT.

## Defining the Fundamentals of Fitness

T cell fitness for CTT remains to be comprehensively defined but mechanisms that determine T cell fitness could include basic T cell functions, receptor and signalling dynamics, metabolic fitness and transcriptional or epigenetic programming. If T cell fitness is to be used a prognostic marker, we need to define biomarker/s that capture T cell functional potential across this broad set of mechanisms and in a broad range of patients.

A range of datasets can be used to identify and refine biomarkers of T cell fitness, including analyses of i) conventional anti-tumour T cell immunity, ii) CTT biology, including correlates in leukapheresis or CAR T cell products, or *in vivo* CAR T cell activity, iii) T cell fitness with ageing, cancer or conventional treatments, and iv) CTT efficacy in patient sub-groups, which will be explored below.

## Lessons From Conventional Anti-Tumour Immunity

T cells play a major role in anti-tumour immunity, with their recruitment to sites of malignancy as tumour infiltrating lymphocytes and targeting of tumour-associated and -specific antigens to mediate effective tumour surveillance. CD8 and CD4 T cells can be divided into antigen-inexperienced naïve T (T_N_) cells and antigen-experienced T cells, which are further subdivided into stem cell memory (T_SCM_), central memory (T_CM_), effector memory (T_EM_), effector memory that re-express CD45RA (T_EMRA_) and effector (T_EFF_) T cells. These phenotypes form a spectrum in terms of proliferative capacity and tumour cytotoxicity. T_SCM_ are highly proliferative and maintain naïve-like properties but are a minor component of peripheral blood (22). T_EFF_ cells are highly cytotoxic and mediate immediate anti-tumour killing but are not robustly proliferative.

Certain T cell phenotypes are associated with reduced anti-tumour immunity [reviewed in (23)]. These include T_EMRA_ cells that lack proliferative capacity (24), exhausted T (T_EX_) cells that have undergone excessive stimulation with antigen and express markers such as PD-1, TIM3 and LAG3 (25), terminally differentiated T (T_TD_) cells that have downregulated both CD28 and CD27 and exhibit markers of DNA damage and cell cycle arrest (26). Senescence phenotypes can also emerge, whereby a cell arrests its cell cycle in response to specific triggers. In T cells, senescence can be driven by excessive cell division, cumulative DNA damage, nutrient stress or inflammatory stress signalling [reviewed in (27)], and often correlates with hyperactivation of kinase signalling pathways that lead to perturbations in T cell receptor (TCR) signalling (28, 29). A predominance of dysfunctional T cells and the induction of senescence is thought to substantially limit anti-tumour immunity (23).

Specific T_EFF_ cell functions are critical for anti-tumour immunity. CD8 T_EFF_ cells can mediate direct cytotoxic killing of tumour cells through the production of cytokines like interferon (IFN)γ, tumour necrosis factor (TNF) superfamily ligands and the release of cytotoxic granules containing perforin and granzymes. CD4 T_EFF_ cells can similarly produce cytokines to limit tumour growth, activate anti-tumour functions of other cells such as macrophages, and augment CD8 T cell responses through provision of CD4 T cell help. CD4 T cell help involves augmenting costimulatory signalling through DCs and/or producing γc cytokines that promote activation and survival (30). It is essential to establish robust CD8 T cell memory populations, support CD8 T cell expansion after re-activation (31) and avoid the induction of T cell exhaustion (32). This has direct effects on anti-tumour immunity, where coincident activation of CD4 and CD8 T cell responses leads to higher magnitude CD8 T cell responses and better clinical outcomes as compared to activating CD8 T cells alone (33); augmenting costimulation appears to be critical for this (32).

Specific metabolic profiles are engaged to support energetically and biosynthetically demanding CD8 and CD4 T cell functions. Engagement of aerobic glycolysis is critical for activated T_EFF_ cells to engage early production of IFNγ (34). High membrane potential in CD8 T cells is also an indicator of subsequent cytokine production capacity (IFNγ and IL-17), while low membrane potential correlates with increased survival capacity (35). Given the complexity of metabolic pathways, multi-parameter profiling techniques are essential for more comprehensive analysis of transitions over the course of both T cell activation and after CTT infusion (36). These functional and metabolic mechanisms also interact with a myriad of transcriptional and epigenetic regulators of T cell activity [reviewed in (37)], to form a substantial pool of potential markers of T cell fitness with anti-tumour immunity.

## Lessons From CTT Biology

With increasing uptake of CTT across a broader array of patients and conditions, T cell characteristics are emerging across the leukapheresis product, the CAR T cell product and after re-infusion that correlate with a successful CTT response (**Figure 1**).

### Markers of CTT Efficacy in Leukapheresis Product

The most desirable prognostic markers of T cell fitness for CTT would be those derived from an untreated or minimally treated patient in the premanufacturing leukapheresis product or isolated PBMCs.

Studies have consistently found that individuals with a higher proportion of proliferative, less differentiated T cell subsets in the leukapheresis product have better CTT outcomes. It was noted early in development of adoptive cell therapy protocols that T_N_ and T_CM_ subsets outperform T_EM_ subsets when used to generate cellular product, despite the fact that T_EM_ cells generated cellular product with greater immediate cytotoxic and cytokine-releasing capacity (38). Fraietta and colleagues closely assessed correlates of CTT efficacy in the leukapheresis product, as well as the CAR T cell product described above (39). Higher frequencies of less differentiated cells, such as T_SCM_ cells or CD8^+^CD45RO^-^CD27^+^ cells, correlated with CRs or partial responses (PR). Indeed, the frequency of CD8^+^CD45RO^-^CD27^+^ cells could be used with a cutoff of 26.5% to discriminate patients with CR and PR or less. Another study by Garfall and colleagues also highlighted that higher frequencies of CD8^+^CD45RO^-^CD27^+^ cells in the leukapheresis product of patients with MM correlated with more proliferation and better CTT responses (40). Increased frequencies of T_N_ (38), T_CM_ (41) and/or T_SCM_ cells (42) appear to correlate with improved engraftment and/or tumour control in adoptive cell transfer models, probably because they have functional advantages compared to more differentiated, less proliferative subsets. For example, T_N_ cells have been shown to possess longer telomeres, more robust proliferative capacity and generate more functional cells (38). Metabolic profiles that support proliferation can also correlate with better CTT efficacy, as is seen in CLL, where reduced Glut1 reserves and therefore reduced glycolytic capacity in PBMCs correlates with poorer outcomes (43).

There may also be markers of T cell fitness in the peripheral blood prior to leukapheresis that are relevant to the leukapheresis product and CTT efficacy. A recent study of NHL and ALL patients noted that CD3^+^ T cell counts in peripheral blood correlated with CD3^+^ T cell yield after leukapheresis which in turn correlated with a higher rate of CR after CTT (44). This highlights the importance of considering whether a patient has undergone, or is undergoing, a treatment that causes lymphodepletion when considering CTT, as this could impact T cell fitness in the leukapheresis product. Indeed, Garfall and colleagues identified that samples that underwent leukapheresis early after diagnosis had greater expansion in the CAR T cell product than R/R MM patients (40).

The CD4:CD8 ratio may also be an indicator of T cell fitness in the leukapheresis product, especially after certain treatments. The normal range for the CD4:CD8 ratio in peripheral blood is between 1.5 and 2.5, but this can vary with age, sex, ethnicity, genetics, exposures, and infections (45). CD4:CD8 ratio declines dramatically with ablative therapies such as autologous stem cell transplantation (ASCT), because CD8 T cells proliferate markedly more than CD4 T cells to reconstitute the T cell compartment after ablation. In R/R MM patients after ASCT, patients that experienced PR or better after experimental CTT tended to have CD4:CD8 ratio in their leukapheresis product that was closer to the normal range (40). Fewer CD4 T cells in the leukapheresis product could result in a lower ratio of CD4:CD8 CAR T cells during manufacturing, which would subsequently restrict CD4 T cell help during reactivation after re-infusion.

### Markers of CTT Efficacy in CAR T Cell Product

Features of the CAR T cell product that correlate with CTT efficacy are also starting to emerge. These correlates could be used during the CTT protocol to advise patients whether they should progress with ablation and reinfusion. Several can be regarded as downstream outcomes of correlates identified in the leukapheresis product.

Firstly, the manufacturing protocol must generate a sufficient number of viable CAR T cells for infusion. This can be a challenge if patients are unable to provide many T cells in their leukapheresis product as discussed above or, potentially, if their T cells are unable to be efficiently activated and expanded. T cells isolated for CTT are typically activated with an anti-CD3 monoclonal antibody (mAb), often in combination with anti-CD28 mAb presented on microbeads. T cell activation, and therefore expansion, is known to be reduced in some scenarios. For example, naïve CD8 T cells from aged mice and humans have an intrinsic defect in T cell signalling that reduces T cell activation and proliferation (29). Similarly, dysfunctional T cell populations, such as T_EX_ cells, are unable to be efficiently activated or proliferate and contribute to the CAR T cell product yield. The number of CAR T cells manufactured is dependent on the proliferative potential of the leukapheresis product, described in the previous section, and can restrict the dose of CAR T cells administered, described in the following section.

Robust T cell activation and proliferation is also needed for efficient CAR transduction with retroviral vectors (46) and it is beneficial, although not essential, for lentiviral vectors. Product derived from T_N_ cells, which have a more robust proliferative capacity, was seen to express higher levels of the tumour-specific receptor following transduction than cell product derived from antigen-experienced T cells (38). This suggests that CAR integration and therefore expression level can be sub-optimal if the leukapheresis product predominantly contains cells with poor proliferative potential. The precise level of CAR expression is variable from cell to cell and patient to patient because lenti- and retroviral transduction systems integrate at random and in variable copy number into the genome. While CAR expression level has not been robustly examined as a correlate of CTT efficacy, recent work suggests that CAR expression at the time of infusion does not strongly correlate with CTT efficacy, although reduced CAR expression after reinfusion can contribute to tumour relapse (47).

The phenotypic profile of CAR T cell product prior to re-infusion may be one of the clearest correlates of CTT efficacy, with transcriptional studies highlighting T cell differentiation and exhaustion as key correlates. Fraietta and colleagues performed an extensive dissection of the composition of the CAR T cell product in CLL patients treated with tisagenlecleucel (tisa-cel) (39). Patients with a CR had transcriptional profiles within the CAR T cell product that were enriched for markers of early memory, suggestive of a higher frequency of less differentiated memory phenotype T cells. CAR T cells from patients with robust CRs also had higher *in vitro* and *in vivo* proliferative capacity and a STAT3/IL6 gene signature that correlated with increased IL-6 production after infusion. Higher expression of exhaustion markers PD-1, TIM3 and LAG3 correlated with PR or NR in the CLL patients (39). In particular, CD8^+^CD27^+^PD-1^-^ cells are a biologically relevant correlate because CAR T cell product depleted of this subset was unable to provide tumour control in a NOD/SCID/γc -/- mouse model. Similarly, a lower T cell exhaustion signature correlated with better CTT efficacy with axicabtagene ciloleucel (axi-cel) and diffuse large B cell lymphoma (DLBCL) in a study by Deng and colleagues (48) and retention of a naïve-like profile with high expression of *Tcf7* as well as a low type I IFN profile correlated with better CTT efficacy in ALL in a study by Chen and colleagues (42). Collectively, these studies demonstrate that low differentiation/high proliferative capacity/low exhaustion cells are optimal for an efficacious CAR T cell product.

The duration of the activation and expansion *in vitro* protocol can also impact on the degree of differentiation in the CAR T cell product, and therefore the CTT efficacy. As a result, abbreviated CAR T cell therapy regimens are being assessed and implemented (49). Curtailed expansion protocols improve efficacy by preventing excessive differentiation and preserving T cell fitness, as well as reducing manufacturing time, although it may require the collection of more leukapheresis material.

The metabolic profile of CAR T cells may also be important. Using sorted cells with lower membrane potential in murine models of adoptive cell therapy leads to greater T cell expansion after transfer and better tumour control (35). Alternatively, using sorted cells with lower glucose uptake in these same models also leads to greater T cell expansion after transfer and better tumour control (50).

Finally, both CD4 and CD8 T cells in the CAR T cell product contribute to CTT efficacy in haematological malignancy. CD8 CAR T cells have more immediate cytotoxic capacity (51), although CD4 CAR T cells have some cytotoxic capacity and are thought to be an important source of IL-2 and other help signals for CD8 CAR T cells during *in vivo* expansion after reinfusion. Accordingly, some CTT products separately manufacture CD4 and CD8 CAR T cells that are then recombined for reinfusion at specific ratios. For example, a 1:1 ratio of CD4 to CD8 CAR T cells proved more effective than CD8 CAR T cells alone in pre-clinical models (51). One such product, now known as lisocabtagene maraleucel (liso-cel), has progressed through clinical studies (51, 52), inducing CRs in 53% of R/R NHL patients in its phase 2 trial, with lower rates of side-effects such as cytokine response syndrome (CRS) and neurotoxicity compared to other forms of CTT (53). Liso-cel has recently been approved for use by the US FDA. Given the growing appreciation of the importance of both CD4 and CD8 CAR T cells (30), any definition of T cell fitness for CTT should evaluate markers in both CD4 and CD8 T cell populations.

Ultimately, cells in the CAR T cell product must be able to i) proliferate in response to TAA, ii) adopt effector functions for killing and iii) differentiate into long-lived memory cells to provide protection against relapse with minimal side effects. It is worth considering whether a particular cell type in the CAR T cell product is able to perform all these functions or whether multiple cell types are needed. Currently, the field favours a model where cells with less-differentiated phenotypes in the CAR T cell product, such as T_N_, T_CM_ and T_SCM_ cells are able to differentiate *in vivo* into both highly cytolytic cells and long-lived memory phenotype cells to support optimal CTT efficacy. CAR T cells undergo a potentially more homogenous differentiation pathway as compared to a normal T cell response, due to their defined manufacturing protocol. Manufacturing protocols can also be used to promote specific fates, such as more potent effector function through the use of IL-2 during culture or a CD28-derived co-stimulatory domain in the CAR, or increased CAR T cell longevity through the use of IL-21 during culture or a 41BB-derived co-stimulatory domain in the CAR (54, 55). Protocols are in development that can guide the generation of specific phenotypes, such as the generation of clinical grade CAR T_SCM_ cells (56). This approach initially enriches for T_N_ cells from the leukapheresis product and includes both the cytokines IL-7 and IL-21 and a small molecule to activate the Wnt/β-catenin pathways during *in vitro* culture. As we understand more about which cells in the CAR T cell product ultimately contribute to specific clinical effects, such as immediate tumour control, memory engraftment and side effects, we may be able to generate more CAR T cell products with a more focused composition.

### Markers of the Optimal T Cell Response in CTT

In order to define novel correlates in leukapheresis and CAR T cell products, it is also important to identify the specific anti-tumour mechanisms engaged after reinfusion during CTT in the patient.

After transfer of CAR T cell product into the patient, CAR T cells aggregate with TAA-expressing malignant cells, which stimulates the CAR receptor and leads to their activation, proliferation and tumour cytotoxicity. Recent work has highlighted that CAR T cells at the point of infusion exhibit an analogous metabolic profile to that seen in T_EFF_ cells early after activation *in vitro* or infection, with peak oxidative and glycolytic marker expression (36). The metabolic profile of CAR T cell product can also be a key correlate of this proliferation, with higher mitochondrial content seen to support more robust expansion after re-infusion and better CTT efficacy (43).

In pre-clinical and clinical studies, the peak magnitude of CAR T cell proliferation and/or the area-under-the-curve (AUC) for early CAR T cell frequency has been seen to correlate with CTT efficacy in terms of the rates of CR or PR for some studies. This may differ based on disease biology, with a correlation seen for paediatric and adult B cell ALL and CLL with tisa-cel and axi-cel (6, 57), but not seen or not as robust for adult DLBCL (4, 58). A key difference here may be the difference in accessibility of malignant cells, which are predominantly freely accessible to newly infused CAR T cells in the blood and bone marrow in leukaemia compared to the less accessible lymph node and extra-medullary sites in lymphoma. Alternatively, accessibility of the proliferating CAR T cells might be more difficult to measure with lymphoma as compared to leukaemia, as proliferation would occur locally at the tumour site and proliferating CAR T cells may not be as easy to sample in the peripheral blood.

Antigen load has also been seen to modestly correlate with proliferation in some studies. Patients with higher tumour burden generated higher numbers of circulating expanded CAR T cells in patients with R/R NHL (59). However, many clinical CAR T cell protocols aim to limit tumour load prior to infusion as higher antigen load can lead to more severe side effects such as tumour lysis syndrome, CRS and neurotoxicity (17) and may accelerate exhaustion. Collectively, this highlights that robust CAR T cell proliferation can enhance CTT efficacy in some scenarios. This proliferation may be constrained by both extrinsic factors (such as antigen accessibility or immunosuppressive environments in the host or tumour [reviewed in (60)], or the intrinsic proliferative capacity of the CAR T cell.

The frequency of CAR T cells after infusion can theoretically be increased by infusing more CAR T cell product into the patient. While higher CAR T cell doses have correlated with improved CTT efficacy in some, but not all, CTT studies (8), increased doses can also result in increased severity of CAR T cell side effects, such as CRS and neurotoxicity (17). CRS, in particular, is thought to be driven by activated CAR T cells with amplification from local monocytes and macrophage, leading to massive release of cytokines including IL-1 and IL-6 (61). This highlights an inherent constraint with CTT: increasing the amount of CAR T cell product infused may, in some applications, increase tumour clearance but it may also increase side effects.

CAR T cells must also engage cytotoxic effector functions. While activated CAR T cells can produce IFNγ and TNF and kill TAA-expressing targets through Fas/FasL-mediated killing (62), the predominant pathway for CAR T cell-mediated clearance of malignant cells is thought to be exocytosis of cytolytic granules (63). CAR T cells form less robust immunological synapses with TAA-expressing targets, which allows them to engage, deliver cytotoxic granule cargo and disengage very rapidly (63). Given the rapidity of CAR engage/disengagement, the level of CAR expression is likely to be important for effective killing, but CAR expression can be down-regulated after TAA-encounter (47). Transient downregulation may be beneficial to avoid exhaustion (64) but more sustained epigenetic-mediated down-regulation of CAR expression has also been shown to lead to relapse in a subset of CLL patients (47).

Finally, some CAR T cells must enter a memory-like state after TAA is cleared to provide ongoing protection against relapse. In CTT clinical studies, patients that do not maintain detectable CAR T cells in peripheral blood can have higher rates of relapse after CTT. For example, a study by Porter and colleagues highlighted that loss of CAR T cell persistence was correlated with poorer disease-free survival in both ALL and CLL (1). However, another study by Park and colleagues saw no significant correlation between CAR persistence and ongoing tumour control in patients that had a complete remission then relapsed with CD19+ malignant cells (16). This distinction may be due to the CAR used, as the Park et al. study used a CAR containing the CD28 domain while the Porter et al. study used a CAR containing the 41BB domain. One way to promote memory formation is to use the 4-1BB co-stimulatory domain in the CAR, which improves persistence of CAR T cells by reducing T cell exhaustion (55, 65, 66).

## Lessons From T Cell Biology

**Figure 2:**
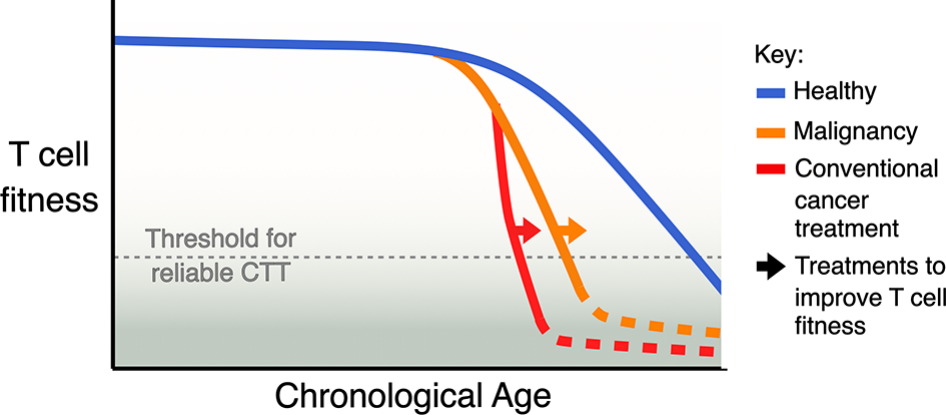
Model of the cumulative impact of ageing, malignancy, and conventional treatment for cancer on T cell fitness. Natural ageing leads to a decline in T cell fitness, malignancy accelerates this decline and some conventional cancer therapies, especially ASCT, can further accelerate this decline. A key aim for improving CTT should be to identify strategies and treatments that can avoid or revert the loss of T cell fitness.

Based on the observations described above (**Figure 1**), our current understanding is that having a high proportion of less differentiated T cells is the key indicator of T cell fitness for CTT in a patient. Less differentiated T cells can differentiate into other populations correlated with high CTT efficacy, such as T_CM_ in the CAR T cell product, activated T_EFF_ after re-infusion, and memory-like subsets to prevent tumour relapse. Less differentiated T cells are also able to proliferate more, to generate a better yield of CAR T cell product and support better expansion after reinfusion. While this broad indicator is helpful, treatment decisions for CTT would benefit greatly from more refined biological markers to define T cell fitness and predict CTT outcomes. We may be able to derive a more accurate biomarker from our understanding of other factors- ageing, chronic infection, malignancy and conventional cancer treatment (**Figure 2**)- that are known to impact T cell function.

### Ageing and T Cell Function

Ageing leads to substantial changes in T cell generation, maintenance, phenotype and function. With increasing age, the relative production of T cell progenitors and their ability to migrate to the thymus decreases (67), and the capacity of the thymus to generate T_N_ cells becomes limited by thymic involution (68). Ageing therefore leads to decreased thymic output, as well as a decreased capacity in peripheral lymph nodes to support new T_N_ cells (69) and an accumulation of less proliferative, antigen-experienced cells with age, which all drive a decrease in the number and proportion of T_N_ cells. This loss of T_N_ cells also restricts the TCR repertoire (70, 71), limiting our ability to respond to new antigens. CD8 T cells are lost preferentially with advanced age (72), leading to an increased CD4:CD8 ratio, although other age-related health conditions such as leukaemia or lymphoma can also perturb this ratio. As a result, an increased CD4:CD8 ratio is a key indicator of broader age-related immune changes that drive a decline in T cell immunity.

Ageing also leads to an accumulation of antigen-experienced and dysfunctional T cell subsets, such as T_EMRA_, T_EX_ and T_TD_ cells, and a selective retention of antigen-inexperienced T cells with memory-like features and NK cell-like markers, known as virtual memory T (T_VM_) cells (29). These cells lose TCR-driven proliferative capacity with age but increase their responsiveness to IL-15 (73) and exhibit enhanced survival capacity. Aged memory phenotype cells also exhibit increased mitochondrial content alongside decreased functional capacity (72). T cells with senescence phenotypes also emerge with advanced age, manifesting in T cells from aged individuals as an increase in levels of γH2Ax (marker of DNA damage), p21 (marker of cell cycle arrest), and Bcl-2 (an antiapoptotic protein that increases the resistance of senescent cells apoptotic cell death), and a reduced proliferative capacity (29). The predominance of non-proliferative, atypical, dysfunctional T cells with age and the induction of varying levels of senescence across all T cell populations is likely to reduce T cell fitness for CTT.

Ageing directly alters the activation capacity of T cells through changes in surface expression of certain receptors and downstream signalling pathways that alter the ability to transmit activation signals. As an example, CD28 is often downregulated on T cells with increasing age, limiting the ability of these T cells to respond to co-stimulatory signals (26). In addition, mitogen activated protein kinase (MAPK) signalling downstream of TCR engagement is perturbed with increasing age and T cell senescence. For example, senescent aged T cells exhibit hyperphosphorylated MAPK, negative regulation of TCR signalling and loss of proliferative capacity (28, 29). In addition, CD8 T cell effector functions change with age. Aged CD8 T cells exhibit a bias towards production of IFNγ and a reduction in production of other cytokines, such as IL-2, higher levels of transcripts for granzymes but a decreased antigen-specific killing capacity (29).

Of note, the normal T cell ageing process can be accelerated or decelerated by host genetics or extrinsic factors, leading to significant variation in the speed of ageing across individuals. As a result, an individual’s chronological age may not correspond with their biological (74) nor, likely, their immunological age. Key factors that can accelerate age-related changes in the T cell population include chronic infection, malignancy and treatments that cause DNA damage or inflammation. Indeed, chronic, low-grade inflammation is known to be a feature of the aged immune system and is termed “inflammaging” (75). It is also becoming clear that chronic inflammation, driven by DNA, metabolic or other forms of cellular damage and an aged immune system, is a dominant driver of the ageing process (76). T cells are especially sensitive to the inflamed aged environment, as young T_N_ cells that a transferred into an aged environment completely lose their TCR-driven proliferative capacity (29), possibly due to kinase hyperactivation and dysregulation of TCR-mediated activation.

### Chronic Infections and T Cell Function

Chronic infections such as human cytomegalovirus (HCMV) or Epstein-Barr virus (EBV) affect around 50-85% of the world’s populations by the age of 40 (77, 78) and the rate of infection increases with age. In healthy individuals, the viruses can persist as latent infections with episodes of reactivation that remain asymptomatic, therefore we presume a large proportion of cancer patients to be seropositive carriers and reactivation could occur with certain treatments like ASCT. Chronic infections also accelerate the accumulation of dysfunctional T cells, presumably by driving replicative senescence and/or exhaustion phenotypes due to the persistence of antigen stimulation. For example, HCMV-specific T cells consist mainly of CD27^+^CD28^-^CD45RA^-^CD45RO^+^ T cells during acute viraemia, but eventually CD27 is lost and CD45RA is re-expressed to generate T_EMRA_ cells (79). Chronic, low levels of immune activation with viral reactivation can also drive low-grade but persistent inflammation to accelerate ageing processes. For example, increased HCMV-specific responses are associated with increased inflammatory markers and age-related diseases (80). This inflammatory milieu could increase senescence in non-antigen-specific T cells.

HCMV can also generate inflationary T cells, which are large oligoclonal HCMV-specific T cell expansions that do not contract but instead remain high or even increase in relative frequency over time (81). Due to these expansions, HCMV-specific T cells comprise at least ~10% of circulating CD4 or CD8 memory T cells in seropositive individuals (82), with higher frequencies seen in older as compared to younger individuals. Inflationary HCMV-specific T cells are, however, metabolically active and broadly functional (83), so it is unclear whether having high proportions of inflationary T cells in leukapheresis product would impact on subsequent CTT efficacy.

### Cancer and T Cell Function

Age-related inflammation can drive age-related T cell dysfunction, but cancer itself may further exacerbate inflammation and compound T cell ageing in patients. High levels of inflammatory cytokines, such as IL-6, are observed both locally and systemically in many haematological malignancies, such as MM and CLL, and this is correlated with poorer prognosis (84).

MM is highly dependent on IL-6 during certain stages of disease progression (84) and increased levels of the highly inflammatory cytokine IL-17 or T helper 17 cells in PBMCs and the bone marrow microenvironment correlates with exacerbated disease (85). In R/R MM, disease-derived inflammation augments γc cytokine signalling pathways and lymphopenia-induced proliferation, to accelerate immuno-senescence (21). This results in a decline in T_N_ cells and metabolic remodelling in the remaining T cells in MM patients. Another study by Suen and colleagues showed that T cell clones from MM patients exhibited a senescent effector phenotype, characterised by upregulation of KLRG1, CD57 and CD160 and downregulation of CD28 (20), highlighting that MM is associated with T cell dysfunction.

CLL has also been associated with premature T cell ageing (86). CLL patients have a decreased CD4:CD8 ratio, which is associated with shorter overall survival in this group (87). T cells from CLL patients also exhibit intrinsic functional deficits; when stimulated *in vitro*, T cells from CLL patients exhibit a significant proliferative deficit when compared to age-matched R/R MM or young adult ALL patients (88). CD8 T cells from patients with CLL are capable of producing cytokines, such as IFNγ and TNF, but their cytotoxic capacity is impaired due to defective granzyme localisation and they express increased levels of PD-1 and other inhibitory receptors (89). This suggests that CLL drives a form of exhaustion where T cells recognise tumour-derived antigen but fail to control disease.

Collectively, this highlights that malignancy and its associated inflammation accelerates the accumulation of age-associated T cell subsets that reduce T cell fitness.

### Cancer Treatment and T Cell Function

Current treatments for haematological malignancies can further accelerate immune ageing (21). Some therapies can alter pathways for repair of DNA damage or protein turnover but one of the most dramatic causes of premature ageing in the T cell compartment is significant lymphopenia caused by drug or radiation exposure [reviewed in (27)]. Ablative cancer therapies are therefore major drivers of T cell ageing in cancer patients.

ASCT is one of these therapies and it is a cornerstone of treatment for haematological cancers, but it perturbs the composition of the T cell compartment and drives substantial metabolic remodelling with significantly increases in mitochondrial membrane potential, thus reducing T cell fitness (21). The shift in composition with ASCT and lymphopenia-inducing therapies is irrecoverable, with permanent increases in T_EM_ and T_EMRA_ cells in MM patients (21). CD4:CD8 ratios can also be perturbed with this treatment, often leading to an inversion in the ratio due to higher CD8 T cells proliferation compared to CD4 T cells in the lymphopenic environment post-transplantation. In children and young adults undergoing ASCT, the CD4:CD8 ratio recovers to normal levels by 1 year post-treatment (90), but in older adults, this ratio takes longer to recover, if at all (90, 91).

Given the individual impacts of ageing, chronic infection, malignancy or treatment on T cell function, we propose a model where the impact of these factors on T cell fitness for CTT is cumulative in older, heavily treated patients (**Figure 2**). A cumulative impact of age, disease and treatment on T cell fitness has been demonstrated in mouse models, which have documented a significant increase in T cell dysfunction (92). It is therefore critical for the field to develop measures of T cell fitness to use as treatment decision aids for older, heavily treated patients with haematological cancers considering CTT.

## Lessons From Clinical CTT Cohorts

T cell fitness is predicted to limit CTT efficacy, and T cell function and phenotype is known to be impacted by age, tumour burden, tumour type and prior treatment, but precise markers for T cell fitness for CTT are yet to be defined. This is, in part, due to a lack of suitable clinical data to identify correlates.

As an example, age-related processes are a major modifier of T cell function and phenotype but our understanding of the impact of these processes on CTT efficacy is currently limited. Recent studies have aimed to explore this point but all have key limitations.

Patient numbers in CTT trials are often too low to detect modest but biologically meaningful differences in efficacy across patient sub-groups. For example, Park and colleagues provided one of the earliest dissections of age and CTT efficacy (16). They reported that efficacy did not differ significantly between 18-30 year old (yo) vs >60 yo cohorts, but efficacy did trend down with increasing age. The authors also noted that the study may not be powered to identify modest differences, with n=14 and n=8 in the younger and older cohorts, respectively.

Definitions used to delimit older and younger cohorts can also reduce our ability to dissect how age-related processes impact CTT efficacy. Recent studies by Neelapu and colleagues and Lin and colleagues have provided some of the largest comparisons of CTT efficacy in younger and older age groups (93, 94). These studies covered two key commercially manufactured CAR T cell products, axi-cel and tisa-cel, and reported that there was no significant difference in CTT efficacy between younger and older cohorts, but these studies had relatively high median ages in their young cohorts (55 and 56 years of age respectively). As such, they do not optimally assess the impact of age-related processes on CTT efficacy in young vs older individuals, as studies of T cell biology generally define young as <30 yo and older as >60 yo (29, 73).

Differences in tumour biology or treatment may also confound analyses. For example, Munshi and colleagues performed a successful clinical trial with idecabtagene vicleucel (ide-cel), showing that older patients with MM had similar or better outcomes than younger patients (8). The composition of the younger vs older cohorts in terms of disease state, prior treatment and CAR T cell dose was unclear from the published data, but MM is predominantly a disease of older individuals, making it difficult to recruit a truly immunologically “young” cohort, and the few young MM patients that can be identified may have more refractory disease that could accelerate a decline in T cell function (95). Similarly, in the study by Neelapu and colleagues, the younger cohort had a higher proportion of patients that had undergone ASCT (30% in younger vs 19% in older) (93). ASCT is known to drive premature biological ageing of the T cell compartment (21) and could further obscure differences in T cell fitness between younger and older patients as defined in this study.

However, it is becoming clear that advanced patient age or disease type can limit T cell isolation, which in turn limits CTT manufacturing. Both increasing patient age and CLL or NHL compared to ALL are significantly associated with reduced lymphocyte collection efficiencies prior to planned manufacture (96). CAR T cell manufacturing can also sometimes fail, with higher rates observed in clinical trials of tisa-cel as compared to other available therapies (97). Presumably T cell fitness could contribute to this and recent work using cells from healthy donors showed that increased age can reduce CAR T cell production, skew CAR T cell phenotype towards more differentiated subsets and potentially reduce CAR expression (98). Recent work has also highlighted potential age-related differences in adverse events. Patients over 65yo reported a higher rate of neuro-toxicity related events and lower rate of CRS events after CTT, with older patients also more likely to have received an out-of-specification product, such as a product with low cell viability (99).

These studies clearly illustrate that advanced age, heavy tumour burden, unfavourable tumour biology and heavy pre-treatment do not preclude the use of CTT; indeed, effective CTT is possible in populations that might be predicted to have poorer T cell fitness. However, we lack tools to accurately identify individual patients who the current protocol is likely to fail, and we may be missing biological insights that could improve therapy outcomes for these patients. It is challenging to recruit large numbers of individuals from both older and younger age groups with comparable cancer biology, disease severity and treatment history, but it will be necessary for meaningful multivariate analyses of CTT efficacy and T cell fitness across different ages, malignancies and treatment groups. Alternatively, a deeper understanding of the fundamental biology of T cell fitness- biological changes in T cells with age, tumour burden and prior treatments described above- could be used to design modifications to CTT for specific patients.

## Developing Aids for Clinical Decision-Making

To ensure the optimal delivery of CTT in the clinic, we need to evaluate whether it delivers real benefit to the patient in a cost-effective manner and biomarkers that can predict the likelihood of outcomes can aid in this. In this cost-efficacy equation, the critical variables are i) the ease of delivery of CTT (i.e. the cost), ii) the likelihood of benefit that that patient receives in terms of clinical responses, and iii) the likelihood of expensive side effects. CTT certainly delivered benefit to a subset of patients in phase I and II studies, which speaks to its biological potential. However, a paucity of randomised controlled trials comparing CTT efficacy and toxicity to conventional treatment strategies has initially left the cost-efficacy question subject to extrapolation and biased assumptions [reviewed in (100)]. The first of these randomised trials are beginning to be reported. ZUMA-7 (CTI: NCT03391466) is comparing CTT with axi-cel to standard-of-care (SOC) ASCT as second-line therapies for DLBCL. BELINDA (CTI: NCT03570892) is a similar study for aggressive B cell NHL using tisa-cel, and TRANSFORM NHL (CTI: NCT03575351) is a similar study using liso-cel. Early reports suggest that axi-cel and liso-cel exhibit enhanced efficacy compared to the current SOC, while tisa-cel has not reached its primary endpoint of event free survival compared to SOC.

In the sections above, we reviewed evidence suggesting that T cell fitness may be a key factor controlling CTT efficacy and there is unequivocal data to show that T cell function is negatively impacted by age, malignancy and some prior therapies in patients. To date, these factors have not been clearly identified as determinants of CTT efficacy *within* a single study, but there is a growing clinical appreciation that they may be determinants by comparing different cohorts *between* studies. This is most clearly demonstrated with tisa-cel therapy in R/R B cell ALL in terms of patient age. If paediatric/adolescent young adult populations are compared with older adult populations, tisa-cel is clearly effective and cost-effective in patients who are <25yo (100), but it is not as effective and probably not cost-effective in patients who are older. T cell fitness in younger patients may play a role in this difference, but other factors such as the poorer cytogenetic and molecular profile that typifies B-ALL in older adults cannot be excluded.

To improve CTT effectiveness for older and/or more heavily-treated patients that currently dominate clinical demand, strategies to optimise T cell fitness should be explored but increased efficacy cannot come at the cost of increased toxicity. In some instances, this has not been achieved. For example, axi-cel appears to have increased response rates compared to tisa-cel therapy in DLBCL and appears to improve event free survival compared to SOC ASCT in the ZUMA-7 trial. However, it also results in higher rates of CTT-associated toxicity, leading to clinical algorithms that favour axi-cel administration to younger patients with fewer comorbidities who are physiologically more resilient (99). In contrast, liso-cel uses a 41BB co-stimulation domain and has maintained its efficacy while reducing its CRS and neurotoxicity profile, resulting in a lower cost of care (53).

**Figure 3:**
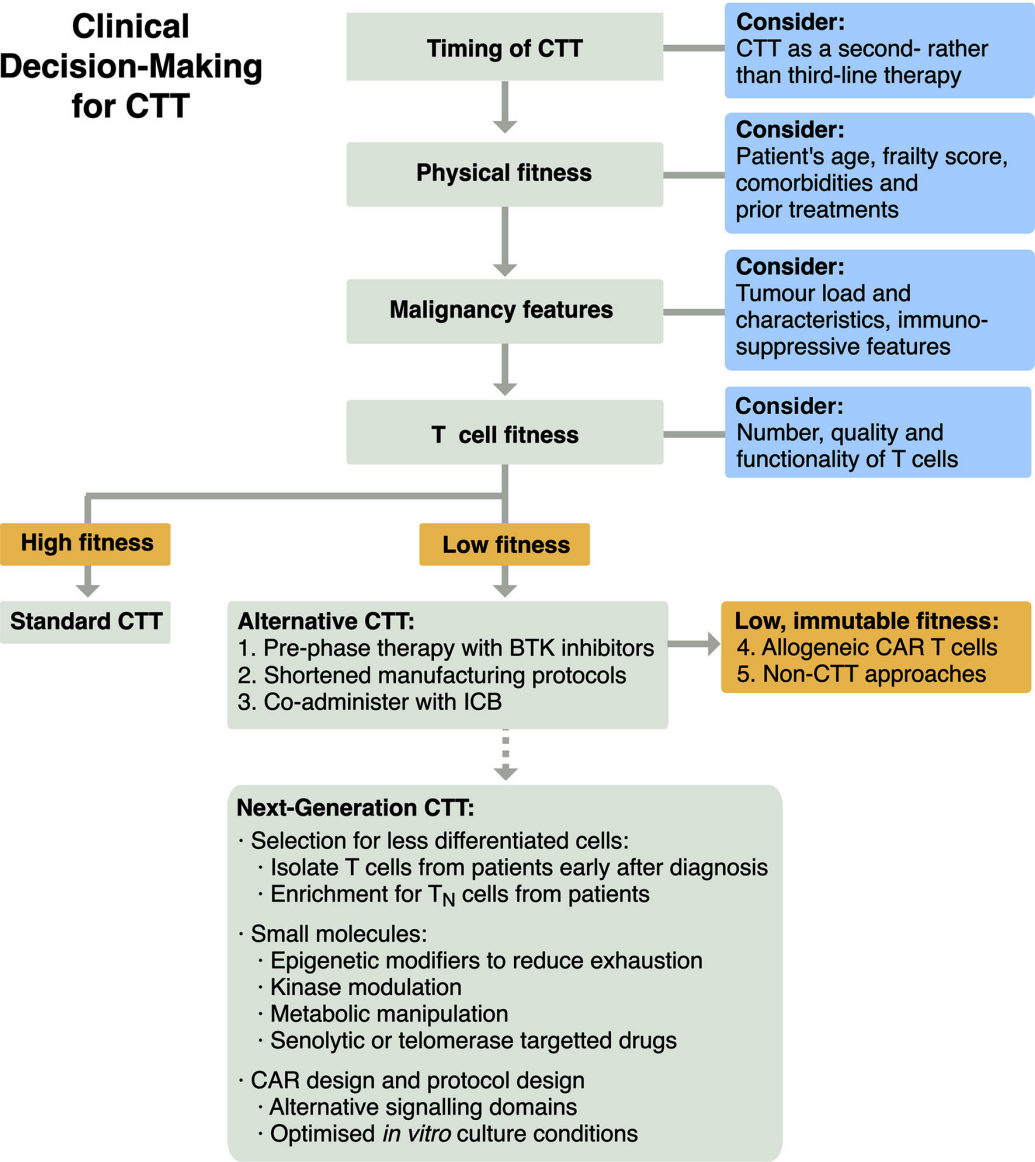
Schema for clinical decision-making for CTT to integrate evaluation of T cell fitness for patients.

To enhance the efficacy of CTT using our current understanding of T cell fitness, a treatment algorithm that incorporates T cell fitness should be formulated (**Figure 3**). Firstly, CTT could be utilised earlier in a patient’s course of treatment, as a second- rather than a third-line therapy. The promising results of axi-cel and liso-cel in ZUMA-7 and TRANSFORM NHL suggest CTT can improve on current SOCs, and earlier intervention may avoid age-, malignancy- and treatment-associated declines in T cell function. Patients considering CTT are currently assessed for their physiological fitness and ability to withstand potential toxicities. Patient assessment could be extended as metrics are developed, to assess i) features of their malignancy that may correlate with efficacy that we have been unable to explore in this review, and ii) their T cell fitness to identify dysfunctional, hypofunctional or senescent phenotypes. Patients with low T cell fitness who also have sufficient time to modify their starting T cell population may be able to pursue *in vivo* pre-phase therapy with Bruton’s tyrosine kinase (BTK) inhibitor therapies, similar to the approach used in trials for brexucabtagene autoleucel (brexu-cel) (88, 101). Alternatively, patients with low T cell fitness may benefit from novel manufacturing strategies, such as the shortened *in vitro* expansion protocol used in brexu-cel (102), or adjunct therapies, such as coadministration with immune checkpoint blockade (ICB) (101, 103). Finally, patients who have low T cell fitness that is immutable may be best served by the delivery of CTT manufactured from healthy allogeneic cell donors (104) or by pursuing therapeutic strategies other than SOC CTT products. This could include both non-CTT approaches and next-generation CTT approaches in development to maximise or recover T cell fitness, as explored below.

## Next-Gen CTT to Increase T Cell Fitness

Additional novel approaches are in development for CTT that could be particularly helpful for patients with low T cell fitness (**Figure 3**).

One straightforward approach to maximise T cell fitness in patients could be to collect and store T cell samples from patients with haematological malignancy immediately at diagnosis. This would be as early as possible with respect to disease progression, prior to any immunomodulating therapies and before the shift in composition in T cell population and phenotype (21), with the aim of collecting higher numbers of T_N_ and T_SCM_ cells. Alternatively, enrichment protocols for less differentiated subsets, such as T_N_ cells, could be used to ensure *in vitro* protocols avoid dysfunctional cells and utilise the fittest cells that can be derived from a patient (51).

We could also include rationally selected drugs, either prior to leukapheresis, into the *in vitro* culture or co-administered with the CAR T cell product to optimise T cell function. For example, recent work has highlighted that exhaustion and CAR downregulation can be reduced by including an epigenetic inhibitor, JQ1, in *in vitro* cultures of CAR T cells from CLL patients (47).

Kinase inhibitors are another approach of interest. BTK inhibitors such as ibrutinib are being used clinically for leukaemia and lymphoma to reduce tumour burden and are now part of the ide-cel CTT protocol, as they correlate with increased efficacy of subsequent CTT (88, 105). T cell dysfunction associated with ageing or inflammation is characterised by promiscuous activation of a number of kinases and CTT protocol modifications that target these pathways could improve outcomes with T cells from older patients. For example, transient MAPK inhibition can reduce hyperactivation and improve the proliferative capacity of aged CD8 T cells in mice (106, 107).

Ageing and prior ASCT are known to lead to metabolic and mitochondrial dysfunction in T cells (21, 74) and a range of metabolic interventions are being explored for CTT. Sorting of metabolically “fit” T cells using dyes for mitochondrial membrane potential can improve adoptive cell therapy outcomes in pre-clinical models, leading to increased expansion after reinfusion and improved tumour control (50). Restraining the engagement of glycolysis, either through genetic modification or the inclusion of glycolysis inhibitors, can also improve these same outcomes (35). Finally, spermidine can be administered *in vitro* and *in vivo* to promote autophagy and mitochondrial turnover in T cells, and it has been seen to improve T cell function in aged mice (108).

Agents that target senescence, known as senolytic drugs, selectively kill senescent cells and can improve tissue function in mouse models of ageing (109). One such drug is Venetoclax, a specific inhibitor of Bcl-2, which induces selective apoptosis in a variety of cells undergoing senescence (110). Venetoclax is approved for clinical use for a variety of haematological cancers and could be used as part of induction therapy prior to CTT. Similarly, transient expression of telomerase using mRNA delivery can improve the proliferative capacity and reduce replicative senescence in CAR T cell products (111).

Tailored CAR design or culturing conditions for patients with low T cell fitness could also be considered. Novel CAR designs with the introduction of costimulatory signalling domains from ICOS, CD27 and other molecules (112, 113) may enhance CTT efficacy even with unfit T cells. Similarly, the inclusion of cytokines such as IL-7, IL-15, and IL-21 into cultures to promote optimal T cell differentiation (56) may be uniquely critical when dealing with patient T cells of low fitness.

A wide range of drugs and treatments that could improve T cell fitness are being considered as viable modifications to *in vitro* and *in vivo* stages of CTT protocols. However, regardless of the strategy to increase T cell fitness, we must ensure that it does not augment CRS and other forms of CTT-associated toxicity.

## Conclusion

Our growing mechanistic understanding of anti-tumour immunity with autologous CTT and markers of CTT efficacy in the leukapheresis and final manufactured product will allow the field to more accurately define T cell fitness for CTT in an individual. The metric of T cell fitness will need to account for a wide range of mechanisms that can be differentially impacted by ageing, chronic infection, cancer and conventional treatments. While this will add complexity to the task of defining a metric for T cell fitness, it also emphasises how T cell fitness will vary from patient to patient. If we can identify accurate measures of T cell fitness in CTT for use as treatment decision-making and tracking tools, we have the opportunity to significantly improve outcomes with this powerful therapeutic, particularly for older, heavily pre-treated patients.

## Author Contributions

All authors contributed to conception and design of the review. PM wrote the first draft of the manuscript and figures. SF, DR, and KQ wrote sections of the manuscript. All authors contributed to manuscript revision, and have read and approved the submitted version.

## Funding

We acknowledge support and funding for this work from an RMIT Research Training Program PhD scholarship (PHM); Bristol Myers Squibb and the Haematology Society of Australia and New Zealand (SF); NHMRC Project Grant ID1161708 (AJ); a Rebecca L. Cooper Foundation Project Grant (ID PG2019500), a Maxwell Eagle Endowment Grant, and an RMIT Vice-Chancellor’s Research Fellowship (KMQ). The funder was not involved in the study design, collection, analysis, interpretation of data, the writing of this article or the decision to submit it for publication.

## Conflict of Interest

SF is a co-inventor on three patents concerning CAR T cell therapy, one of which is licensed with royalties paid from Bristol Myers Squibb (BMS) and is a co-principal investigator on a sponsored research agreement investigating CAR T cell therapy with BMS. RK and DR receive research funding from CRISPR Therapeutics.

The remaining authors declare that the research was conducted in the absence of any commercial or financial relationships that could be construed as a potential conflict of interest.

## Publisher’s Note

All claims expressed in this article are solely those of the authors and do not necessarily represent those of their affiliated organizations, or those of the publisher, the editors and the reviewers. Any product that may be evaluated in this article, or claim that may be made by its manufacturer, is not guaranteed or endorsed by the publisher.

## Abbreviations

ALL, acute lymphocytic leukaemia; ASCT, autologous stem cell transplantation; AUC, area-under-the-curve; axi-cel, axicabtagene ciloleucel; brexu-cel, brexucabtagene autoleucel; BTK, Bruton’s tyrosine kinase; CAR, chimeric antigen receptor; CTT, chimeric antigen receptor T cell therapy; CLL, chronic lymphocytic leukaemia; CR, complete responses; CRS, cytokine release syndrome; DLBCL, diffuse large B cell lymphoma; EBV, Epstein-Barr virus; FDA, Food and Drug Administration; ICB, immune checkpoint blockade; ide-cel, idecabtagene vicleucel; IL, interleukin; IFN, interferon; HCMV, human cytomegalovirus; liso-cel, lisocabtagene maraleucel; mAb, monoclonal antibody; MAPK, mitogen activated protein kinase; MM, multiple myeloma; NHL, Non-Hodgkin lymphoma; NR, non-response; PBMC, peripheral blood mononuclear cell; PR, partial response; R/R, relapsed/refractory; SOC, standard-of-care; tisa-cel, tisagenlecleucel; T_CM_, central memory T cell; TCR, T cell receptor; T_EFF_, effector T cell; T_EM_, effector memory T cell; T_EMRA_, effector memory that re-express CD45RA T cell; T_EX_, exhausted T cell; T_N_, naïve T cell; T_SCM_, stem cell memory T cell; T_TD_, terminally differentiated T cell; T_VM_, virtual memory T cell; TAA, tumour associated antigen; TNF, tumour necrosis factor; yo, year old.
